# Hepatitis-E-Virus-Infektion bei einem Patienten mit rheumatoider Arthritis unter Baricitinib-Therapie

**DOI:** 10.1007/s00393-021-01020-4

**Published:** 2021-06-07

**Authors:** Larissa Valor-Méndez, Bernhard Manger, Georg Schett, Arnd Kleyer

**Affiliations:** 1 Medizinische Klinik 3, Rheumatologie und Immunologie, Friedrich-Alexander-Universität Erlangen-Nürnberg und Universitätsklinikum Erlangen, Ulmenweg 18, 91054 Erlangen, Deutschland; 2 Deutsches Zentrum für Immuntherapie (DZI) FAU Erlangen-Nürnberg und Universitätsklinikum Erlangen, Erlangen, Deutschland

**Keywords:** Hepatitis-E-Virus (HEV), Infektion, Rheumatoide Arthritis, Baricitinib, Januskinase-1/2-Inhibitor, Hepatitis E virus (HEV), Infection, Rheumatoid arthritis, Baricitinib, Janus kinase 1/2 inhibitor

## Abstract

Es wurde ein Patient mit rheumatoider Arthritis (RA) vorgestellt, der unter der Therapie mit dem Januskinase-1/2-Inhibitor Baricitinib eine Infektion mit dem Hepatitis-E-Virus (HEV) entwickelte. Unser Patient hatte bei seiner vierteljährlichen Routineuntersuchung deutlich erhöhte Transaminasen bei einer unauffälligen körperlichen Untersuchung. Es fanden sich Antikörper der IgM- und IgG-Klasse gegen HEV und ein erhöhtes C‑reaktives Protein (CRP) sowie HEV-RNA mittels Real-Time-PCR, was auf eine frische HEV-Infektion hinwies. Baricitinib wurde sofort abgesetzt. Die ausführliche Anamnese ergab, dass der Patient Tage vor der Konsultation Rindertartar verzehrt hatte, ohne dass gastrointestinale Symptome oder Fieber aufgetreten waren. Im weiteren Verlauf erholte sich der Patient vollständig, und die Leberfunktionstests und das CRP normalisierten sich innerhalb von 3 Monaten. Baricitinib wurde daraufhin wieder eingenommen. Bisher wurden nur wenige Daten zu HEV-Infektionen bei RA-Patienten, die mit JAK-Inhibitoren behandelt wurden, publiziert.

## Abstract (en)

A patient with rheumatoid arthritis (RA) was presented, who developed an infection with the hepatitis E virus (HEV) under treatment with the Janus kinase (JAK) 1 and 2 inhibitor baricitinib. In the 3‑month routine check-up the patient had clearly elevated transaminase levels with an inconspicuous physical examination. The investigations detected antibodies of IgM and IgG classes against HEV and an elevated C‑reactive protein (CRP) level as well as HEV-RNA by real-time PCR, which is indicative of a recent HEV infection. Baricitinib was immediately discontinued. The extensive anamnesis revealed that the patient had eaten beef tartar some days before the consultation, without the occurrence of gastrointestinal symptoms or fever. In the further course the patient completely recovered and the liver function tests and the CRP levels normalized within 3 months. Baricitinib was then restarted. So far only few reports have been published on HEV infections in RA patients who have been treated with JAK inhibitors.

Eine Leberbeteiligung ist eine der häufigsten Komplikationen der immunsuppressiven Therapie bei Patienten mit rheumatoider Arthritis (RA). Ein Rezidiv von Hepatitis-B- und -C-Viren oder eine De-novo-Infektion der Leber kann als Nebenwirkung von synthetischen/zielgerichteten synthetischen oder biologischen krankheitsmodifizierenden Antirheumatika auftreten [1, 2]. Hier wird über einen RA-Patienten berichtet, der unter der Behandlung mit Baricitinib eine Hepatitis-E-Virus(HEV)-Infektion entwickelte. Baricitinib ist ein Januskinase-1/2(JAK1/2)-Inhibitor, der zur Therapie der rheumatoiden Arthritis (RA) zugelassen ist [3].

## Fallbericht

Ein 71-jähriger Mann, bei dem 2016 eine seropositive rheumatoide Arthritis (RA) diagnostiziert wurde, wurde seit September 2019 mit Baricitinib in einer Dosis von 4 mg/Tag behandelt. Zuvor hatte er 1 Jahr lang Methotrexat in Dosierungen zwischen 10 und 15 mg/Woche erhalten, das wegen erhöhter Kreatininwerte abgesetzt werden musste. Baricitinib war gut wirksam, und der Patient befand sich in anhaltender Remission seiner RA. Die Anamnese umfasste eine arterielle Hypertonie, Hyperurikämie, Dyslipidämie und Nikotinabusus mit Febuxostat und Atorvastatin als Begleitmedikation. Im Jahr 2015 hatte er einen positiven Quantiferon(QT)-Test und deswegen eine 9‑monatige Prophylaxe mit Isoniazid erhalten. Im Jahr 2019, vor dem Beginn der Behandlung mit Baricitinib, war sein QT-Test negativ.

Unser Patient stellte sich in unserem Krankenhaus zu seiner vierteljährlichen Routineuntersuchung vor. Die Anamnese und körperliche Untersuchung ergaben keine Anomalitäten. Er hatte keine Anzeichen einer aktiven RA. Die Leberfunktionstests ergaben einen AST-Wert von 1265 IU/l, einen ALT-Wert von 2112 IU/l, einen LDH-Wert von 553 IU/l und einen GGT-Wert von 261 IU/l (Normalbereiche: > 35 IU/l, > 35 IU/l, < 250 IU/l bzw. 40 IU/l). Auch das CRP war mit 25,4 mg/l erhöht (Normalbereich < 5 mg/l). Zunächst wurde angenommen, dass diese Veränderungen mit der Medikation zusammenhängen; daher wurden Baricitinib, Febuxostat und Atorvastatin sofort abgesetzt. Überseereisen, Alkoholmissbrauch oder die Einnahme möglicher hepatotoxischer Substanzen wurden verneint. Eine mögliche Autoimmunhepatitis wurde ausgeschlossen.

**Figure 1:**
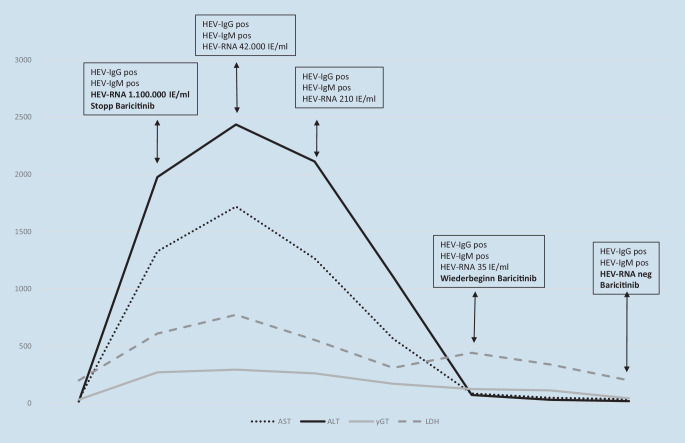

Real-time-PCR-HEV-RNA und HEV-IgM/IgG waren positiv, was auf eine frische HEV-Infektion hinweist. Die Abdomensonographie zeigte keine Hinweise auf eine Schädigung des Leberparenchyms oder der Perfusion. Fünf Tage später begann sich die Leberfunktion zu verbessern und zeigte eine AST von 564 IU/l, eine ALT von 1106 IU/l und eine GGT von 170 IU/l. Im weiteren Verlauf erholte sich der Patient vollständig. Die Leberfunktionstests und das CRP normalisierten sich innerhalb von 3 Monaten. Die Abb. 1 demonstriert den Verlauf der Labortests. Extrahepatische Manifestationen wie neurologische Symptome, eingeschränkte Nierenfunktion oder Kryoglobulinämie wurden nicht gefunden. Nach 3 Monaten war die PCR-HEV-RNA wieder negativ. Daraufhin wurde die Therapie mit Baricitinib wieder begonnen. Während der weiteren 5 Monate der Nachbeobachtung entwickelten sich keine weiteren klinischen oder Laboranomalien. Die RA blieb trotz der 3‑monatigen Unterbrechung der Baricitinib-Behandlung in Remission.

## Diskussion

HEV ist ein einzelsträngiges RNA-Virus, das hauptsächlich die Leber infiziert [4]. Die Infektion erfolgt über die Aufnahme von kontaminiertem Wasser oder Lebensmitteln. Insbesondere rohes oder unzureichend gegartes Fleisch kann eine HEV-Infektion auslösen. Tatsächlich ergab die ausführliche Anamnese, dass der Patient nur wenige Tage vor der Konsultation Rindertartar gegessen hatte, ohne unmittelbare gastrointestinale Symptome oder Fieber zu zeigen. HEV ist die häufigste akute Virushepatitis und gilt derzeit weltweit als wichtiges Problem der öffentlichen Gesundheit; in den westlichen Ländern betrifft sie v. a. immungeschwächte Patienten. In Europa werden 5–15 % der akuten Hepatitiden unbekannter Herkunft durch HEV verursacht [4–6]. Akute Infektionen sind meist asymptomatisch, selbstlimitierend und erfordern keine antivirale Behandlung. Allerdings kann HEV bei immungeschwächten Patienten chronisch werden, z. B. bei Patienten, die eine immunsuppressive Therapie erhalten oder eine hämatologische Erkrankung aufweisen [6–8], auch wenn die Baricitinib-Therapie bei unserem Patienten nicht als direkte Ursache der HEV-Infektion nachgewiesen werden kann. Es wurde berichtet, dass einige symptomatische HEV-Infektionen als medikamentös induzierte Leberschäden fehldiagnostiziert werden können [5, 6]. Eine HEV-Infektion kann insbesondere bei schwangeren Frauen eine fulminante Hepatitis mit einer Sterblichkeitsrate von bis zu 30 % verursachen. Darüber hinaus kann HEV vertikal von infizierten Müttern auf ihre Säuglinge übertragen werden mit erheblicher perinataler Morbidität und Mortalität [9].

Vor der Baricitinib Therapie hatte unser Patient keinen Hinweis auf eine Infektion mit Hepatitis-A-, -B- oder -C-Viren gemäß den bekannten empfohlenen Diagnostikrichtlinien des Robert Koch-Instituts vor immunmodulierenden Therapien. Hier würde sicherlich auch die Durchführung einer HEV-Antikörperbestimmung sinnvoll sein. Die Untersuchung von früher eingefrorenen Serumproben bestätigte, dass die HEV-Infektion nicht auf einer Reaktivierung beruhte, sondern eine De-novo-Infektion war. In früheren Arbeiten wurde eine HEV-Infektion bei mit Tocilizumab behandelten RA-Patienten beschrieben, während zu JAK-Inhibitoren bisher nur wenige Daten veröffentlicht sind [8]. Sowohl Tocilizumab als auch JAK-Inhibitoren blockieren die Wirkung von IL‑6, von dem bekannt ist, dass es Lebersynthesevorgänge bei der die Akute-Phase-Reaktion und beim Eisenstoffwechsel steuert [10]. Unter Tocilizumab und JAK-Inhibitoren wurde über eine Erhöhung der Leberenzyme berichtet [3]. Darüber hinaus zeigte eine aktuelle Publikation, dass die zelluläre angeborene Immunität gegen HEV interessanterweise teilweise von der JAK-STAT-Kaskade der IFN-Signalisierung abhängig ist [11]. Wir denken daher, dass es in unserem Fall wichtig war, die Baricitinib Therapie zu stoppen, um eine adäquate HEV-Abwehr und Clearance zu ermöglichen. Die Therapie wurde alsbald – als die Leberenzymerhöhung sich normalisiert hatte – wieder begonnen. Dieser Fall zeigt auch, dass eine HEV-Infektion eine wichtige Differenzialdiagnose für akute Leberenzymerhöhungen bei RA-Patienten ist, die mit Antirheumatika behandelt werden, und in die Abklärung solcher Ereignisse einbezogen werden sollte.

## Fazit für die Praxis

1. Leberenzymerhöhungen sollten immer ernst genommen werden, sowohl von den behandelnden Rheumatologen als auch von Hausärzten und Patienten.
2. Bei Leberenzymerhöhungen sollten hepatoaffine Infektionen und cholestatische Pathologien zunächst ausgeschlossen werden. Dies gilt insbesondere dann, wenn immunmodulierende Therapien über einen langen Zeitraum eingenommen/angewendet wurden, ohne dass signifikante Leberenzymerhöhungen auftraten, bevor wir diese als durch die immunmodulierende Therapien verursacht bezeichnen.
